# Mitoxantrone hydrochloride liposome-based chemotherapy plus rituximab in elderly patients older than 80 years with diffuse large B-cell lymphoma: case report and review of the literature

**DOI:** 10.3389/fmed.2025.1629168

**Published:** 2025-10-20

**Authors:** Jin-Ping Pi, Ying Liu, Jun Jin, Wei Zhang, Xiao-Hui He

**Affiliations:** ^1^Department of Medical Oncology, Beijing Chaoyang District Sanhuan Cancer Hospital, Beijing, China; ^2^Department of Nuclear Medicine (PET-CT Center), Cancer Hospital, Chinese Academy of Medical Sciences, Beijing, China; ^3^Department of Medical Oncology, Cancer Hospital, Chinese Academy of Medical Sciences, Beijing, China

**Keywords:** diffuse large B-cell lymphoma, case report, mitoxantrone hydrochloride liposome, cardiac involvement, older patients

## Abstract

**Background:**

Treating diffuse large B-cell lymphoma (DLBCL) in patients over 80 years of age remains a significant clinical challenge due to the unfavorable biology of the disease, worse baseline health status (such as cardiac dysfunction), and the toxicities associated with cytotoxic chemotherapy. In this context, attenuated regimens may provide a more favorable risk–benefit profile for elderly DLBCL patients. Mitoxantrone hydrochloride liposome, a liposomal formulation of the synthetic anthracycline anticancer drug, offers several inherent advantages, including reduced cardiotoxicity and targeted delivery to tumor tissue, leading to improved antitumor efficacy and minimized systemic toxicity.

**Case presentation:**

Therefore, we report a case-series study evaluating the efficacy and safety of mitoxantrone hydrochloride liposome-based chemotherapy plus rituximab in three patients with DLBCL aged over 80 years. Two patients achieved complete metabolic remission (CMR) after treatment with mitoxantrone hydrochloride liposome-based regimens, while one attained CMR after receiving this first-line chemotherapy regimen combined with local radiotherapy. Additionally, the safety profile was acceptable and manageable.

**Conclusion:**

These cases suggest that mitoxantrone hydrochloride liposome-based chemotherapy may be effective (all three cases achieved CMR) in very elderly DLBCL patients, warranting further evaluation in larger cohorts.

## 1 Introduction

Diffuse large B-cell lymphoma (DLBCL) is the most common form of lymphoma (1), and often occurs in older persons, with a median age of 66 years at diagnosis (2). Rituximab-CHOP (cyclophosphamide, doxorubicin, vincristine, and prednisone) or rituximab-CHP-polatuzumab is the standard of care for fit patients without cardiac contraindications (3, 4). However, patients aged over 80 years have a poorer prognosis, which may result from an interplay of unfavorable biology of the disease (such as double expression of *MYC* and *BCL2*), baseline health status (impaired cardiac function), suboptimal management, and late toxicities of chemotherapy (2, 5–7). Improving the prognosis for this patient population remains an unmet need.

Mitoxantrone, a synthetic anthracycline anticancer drug, is one of the commonly utilized chemotherapeutic agents in lymphoma, and its substitution for doxorubicin in the R-CHOP regimen has been applied in DLBCL (8, 9). However, cardiotoxicity associated with conventional anthracyclines is a major concern for patients, particularly in older patients (10). Emerging evidence suggests that liposomal anthracyclines offer a promising solution to mitigate this cardiotoxicity concern (11). Mitoxantrone hydrochloride liposome is a novel formulation that was marketed in China in 2022, which holds distinct advantages due to its ability to enhance drug stability, sustain release, and target tumor tissues (12). Preclinical studies suggest that liposomal mitoxantrone may reduce cardiotoxicity, potentially due to a lower peak concentration, larger area under the concentration-time curve, prolonged half-life, and reduced distribution in cardiac tissues (13–15). Meanwhile, clinical trials have demonstrated that many fewer adverse events (AEs) occur in cancer patients treated with mitoxantrone hydrochloride liposome when compared to those receiving conventional mitoxantrone (13). Therefore, we reported a case-series study of three older patients aged over 80 years with DLBCL who received mitoxantrone hydrochloride liposome-based chemotherapy and rituximab to evaluate their efficacy.

## 2 Case presentation

### 2.1 Case 1

An 81-year-old female (Supplementary Table S1) with a weight of 42 kg, a body surface area (BSA) of 1.43 m^2^, and a Karnofsky Performance Status (KPS) score of 80 was classified as healthy based on a simplified geriatric assessment (sGA). Her medical history included a 20-year history of hypertension and previous pelvic radiation therapy. She had a history of DLBCL with two relapses, previously treated with anthracycline-based regimens (CHOP and R-CHOP), achieving long-term remission.

**Figure 1:**
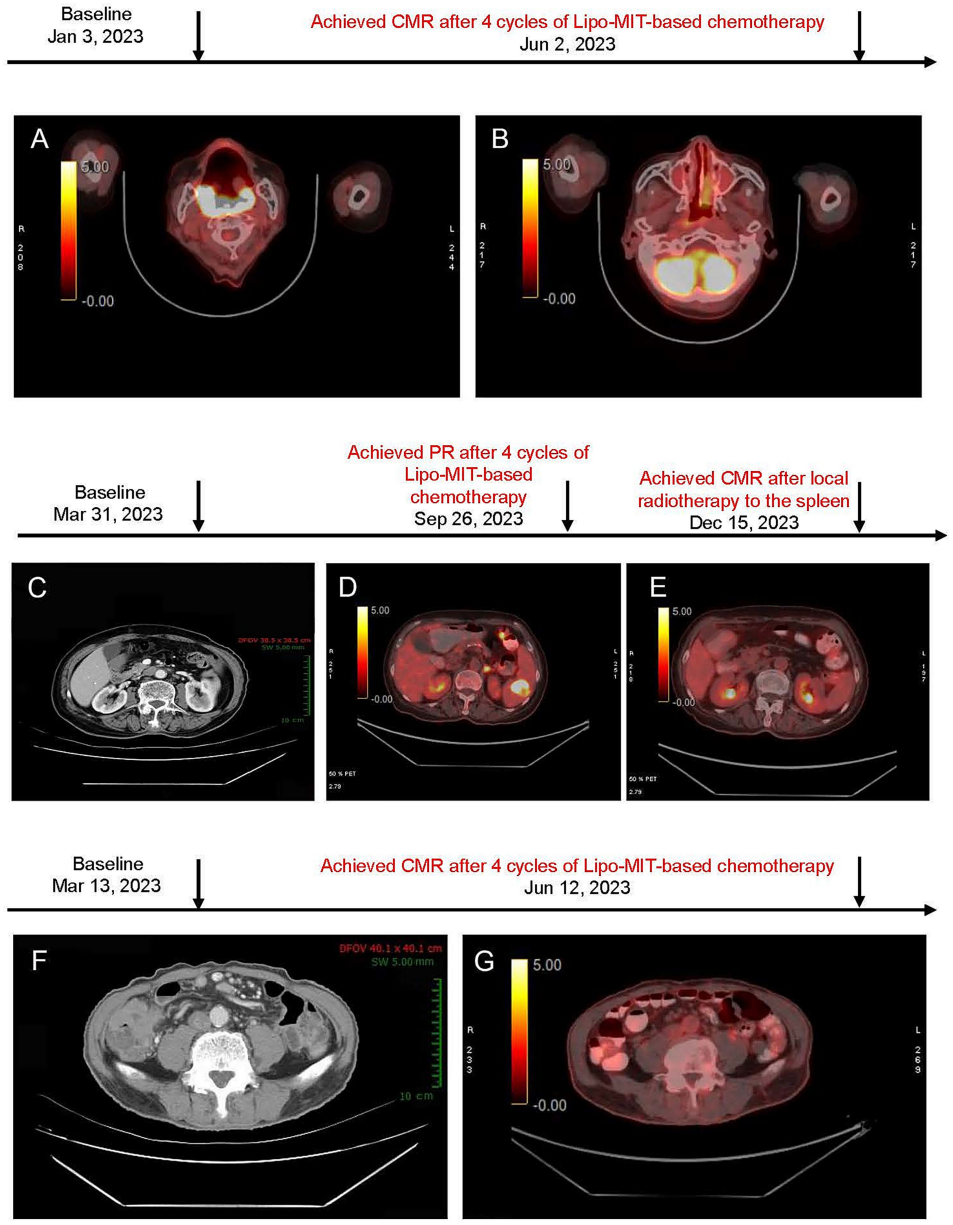
**(A,B)** PET/CT images before and after mitoxantrone hydrochloride liposome-based chemotherapy for case 1. **(A)** Baseline PET/CT image (January 3, 2023) showed soft tissue thickening in the right nasopharynx, soft palate, bilateral oropharynx, base of tongue, and the right parapharyngeal space. **(B)** Post-treatment PET/CT image (June 2, 2023) demonstrated that the right retropharyngeal group and bilateral deep cervical chains were observed with multiple lymph nodes (no increased standardized uptake value, and maximum short-axis diameter approximately of 0.5 cm). Deauville score = 3. **(C–E)** Imaging findings of the neck, chest, abdomen, and pelvis in case 2. **(C)** Baseline CT scan (March 31, 2023) revealed a Splenic mass (~ 3.2 × 2.8 cm). **(D)** After six cycles of treatment (September 26, 2023), PET/CT showed low-density lesion within the spleen (~1.6 × 1.0 cm) and increased uptake (maximum SUV of 8.5) were observed, suggesting the presence of residual tumor (Deauville score = 5). This patient achieved PR. **(E)** Follow-up PET/CT (December 15, 2023) demonstrated further reduction in lesion size, a slightly low density in the spleen with diminished size compared to the previous assessment, and no obvious signs of increased metabolic activity (Deauville score = 1). This suggests an improvement post-treatment. **(F–G)** Imaging findings of neck, chest, abdomen, and pelvis for case 3. **(F)** Baseline CT scan (March 13, 2023) showed significant thickening of the ileocecal wall (maximum SUV 2.1 cm). Surrounding lymph nodes were observed (maximum short-axis diameter ~ 0.8 cm); **(G)** Post-treatment PET/CT scan (Jun 12, 2023) revealed the thickening of the ileocecal wall with diffuse uptake (maximum SUV of 3.2); multiple lymph nodes around were identified with no obvious increased uptake (maximum SUV of 2.2; maximum short-axis diameter ~ 0.5 × 0.5 cm; Deauville score = 3).

In January 2023, she presented with recurrent DLBCL involving the right nasopharynx and cervical lymph nodes, confirmed by PET/CT [maximum standardized uptake value (SUVmax) 31.5] and biopsy as stage IV disease (Figure 1A). The echocardiogram revealed a calcified posterior mitral valve, mild mitral regurgitation, left ventricular diastolic dysfunction (grade 1), and a premature beat; the left ventricular ejection fraction (LVEF) was 67% (Table 1). Baseline blood tests revealed leukopenia [white blood cell (WBC) 2.86 × 10^9^/L] and anemia (hemoglobin 91 g/L; Supplementary Table S2).

**Table 1:** The change in safety data during the treatment.

Treatment course	Case 1				Case 2				Case 3			
	LVEF (%)	NT-proBNP, pg./mL	Cardiac safety		LVEF (%)	NT-proBNP, pg./mL	Cardiac safety		LVEF (%)	NT-proBNP, pg./mL	Cardiac safety	
			ECG	Echo			ECG	Echo			ECG	Echo
Pre-treatment	61	566	SR, SA, frequent APBs, non-PAT	Calcified posterior mitral valve, mild mitral regurgitation, grade I diastolic dysfunction, and premature beat	66	84.8	Complete LBBB	Grade II diastolic dysfunction	74	39.6	LBBB	Mild regurgitation in both the mitral and aortic valves with grade 1 left ventricular diastolic dysfunction
Cycle 2	NE^*^	729	NE^*^	NE^*^	65	79.3	NE^*^	NE^*^	NE^*^	84.7	NE^*^	NE^*^
Cycle 4	64	568	NE^*^	NE^*^	63	81.5	NE^*^	NE^*^	NE^*^	NE^*^	NE^*^	NE^*^
Post-treatment	68	374	SR, supra-VPBs, mild QT interval prolongation	Mild calcification and reflux in the posterior mitral valve leaflet, mild tricuspid regurgitation, and grade I left ventricular diastolic dysfunction	62	84	Complete LBBB, ST-T changes	Aortic valve calcification, mild mitral/tricuspid regurgitation, and grade II left ventricular diastolic dysfunction	62	50.7	LBBB	Mild mitral regurgitation, mild aortic regurgitation, and grade I left ventricular diastolic dysfunction

LVEF, left ventricular ejection fraction; SR, sinus rhythm; SA, sinus arrhythmia; APBs, atrial premature beats; PAT, paroxysmal atrial tachycardia; VPB, ventricular premature beat; LBBB, left bundle branch block; ECG, electrocardiogram; Echo, echocardiogram. ^*^NE indicates parameters that were not evaluated, due to the retrospective study nature.

Given her prior anthracycline exposure and having reached the maximum cumulative dose, the patient initiated a mitoxantrone hydrochloride liposome-based regimen on January 31, 2023. She underwent six 21-day cycles of a mitoxantrone hydrochloride liposome-based chemotherapy regimen. This regimen included mitoxantrone hydrochloride liposome (10 mg on day 1), cyclophosphamide (0.6 g on day 1), prednisone (50 mg on days 1–5), and rituximab (500 mg on day 0); vincristine was discontinued after four cycles due to neurotoxicity.

A PET/CT scan after two cycles showed a remarkable reduction in tumor size and confirmed complete metabolic remission (CMR) according to Lugano 2014 criteria at four cycles (Figure 1B). The progression-free survival (PFS) and duration of response (DoR) were both 11 months.

In this case, the treatment was generally well tolerated. The patient experienced grade 2 anemia, grade 2 neutrophil count decreased, and grade 2 gastrointestinal symptoms. Additionally, grade 1 peripheral nerve infection and grade 1 myelosuppression were observed (Supplementary Table S3). All AEs were mild in severity and managed successfully with supportive care, including Shengbai oral liquid (commonly used for the treatment of leukopenia in clinical practice) and long-acting granulocyte-colony stimulating factor. Regarding the cardiac safety (Table 1 and Supplementary Table S4), echocardiogram during treatment indicated stable cardiac function [only observed mild calcification and reflux in the posterior mitral valve leaflet, mild tricuspid regurgitation, and left ventricular diastolic dysfunction (grade I)]. No obvious changes in the LVEF were observed between the pre- and post-treatment assessments. Electrocardiographic findings remained generally stable. In measurements of NT-proBNP, a cardiac biomarker indicator, the NT-proBNP values remained within the normal range during the treatment. Unfortunately, the patient died from coronavirus disease 2019 (COVID-19) in December 2023.

### 2.2 Case 2

An 82-year-old female (Supplementary Table S1), weighing 52 kg, with a BSA of 1.52 m^2^, and a KPS of 80, classified as fit based on sGA, presented at Sanhuan Cancer Hospital on March 31, 2023. She was diagnosed with Stage III DLBCL according to the Lugano 2014 staging system [non-germinal center B-cell-like (non-GCB), international prognostic index (IPI score = 2)]. CT imaging revealed a splenic lesion (3.2 × 2.8 cm) and multiple lymph node involvements, including supraclavicular, axillary, mediastinal, pulmonary hilum, and retroperitoneal areas (Figure 1C).

From April 1, 2023, the patient received six cycles of mitoxantrone hydrochloride liposome-based chemotherapy (vincristine and cyclophosphamide) plus rituximab. Prednisone was not administered due to gastrointestinal intolerance. Treatment modifications included withholding chemotherapy in cycle 4 due to rituximab-induced fever, and discontinuation of cyclophosphamide in cycles 5–6 owing to persistent myelosuppression.

The patient achieved partial remission after four cycles (Figure 1D), and disease progression was noted at the splenic site at cycle 6. Finally, after local radiotherapy to the spleen for a month, she achieved CMR confirmed by PET/CT (Figure 1E). The PFS and DOR were 22 months and 14 months, respectively. The patient remained in complete remission as of June 13, 2024. However, disease progression was observed by February 2025 during follow-up evaluations, and subsequent second-line treatment with the POLA-BR regimen (polatuzumab vedotin, bendamustine, and rituximab) was performed according to the guideline. The treatment is ongoing.

During the treatment, the patient experienced grade 2 gastrointestinal and hematologic toxicities, including grade 2 anemia, grade 2 neutropenia, and grade 1 myelosuppression, which were generally manageable (Supplementary Table S3). Laboratory tests before and after treatment remained within normal ranges (Supplementary Table S2). Supportive treatments included Shengbai oral liquid and inosine (support recovery from bone marrow suppression). Initial echocardiogram revealed grade II diastolic dysfunction, which improved to grade I (cycle 3), and then she developed aortic valve calcification, mild mitral/tricuspid regurgitation, and grade II left ventricular diastolic dysfunction (post-treatment). However, pre- and post-treatment assessments demonstrated no obvious change in LVEF (Table 1 and Supplementary Table S4). Electrocardiographic results showed no significant changes throughout the treatment. The NT-proBNP values remained normal during the treatment (Table 1).

### 2.3 Case 3

An 82-year-old male (weight of 50 kg, BSA of 1.55 m^2^, and KPS scales of 80, classified as fit based on sGA; Supplementary Table S1) with a history of diabetes (Type 2) managed with oral acarbose presented at Sanhuan Cancer Hospital on March 13, 2023.

He presented with stage II DLBCL involving the ileocecal region and peripheral lymph nodes. The tumor exhibited double expression of *MYC* and *BCL2*. Baseline PET/CT showed markedly increased metabolic activity in the thickened ileocecal intestinal wall (SUVmax 23.2) with associated small lymphadenopathy (Figure 1F). Furthermore, his echocardiogram demonstrated mild regurgitation in both the mitral and aortic valves with left ventricular diastolic dysfunction (grade 1); the LVEF was 74% (Table 1).

On March 14, 2023, the patient initiated receiving six cycles of 3-weekly mitoxantrone hydrochloride liposome (did not perform at cycle 1 due to concerns of demyelinating encephalopathy)-based chemotherapy (vincristine and cyclophosphamide) plus rituximab.

The patient reached PR at cycle 2 and achieved CMR at cycle 4 (Figure 1G; June 12, 2023). As of June 13, 2024, the patient remained in complete remission, and at the most recent follow-up in March 2025, there was no evidence of disease recurrence. The PFS was over 28 months, and the DoR was 24 months.

Throughout treatment, grade 2 anemia and grade 2 neutrophil count decreased were observed (Supplementary Table S3). Laboratory tests before and after treatment remained within normal ranges (Supplementary Table S2). Echocardiograms showed mild mitral regurgitation, aortic regurgitation (mild), and left ventricular diastolic dysfunction (Grade I); stable LVEF values (62%) were also observed. Electrocardiograms before and after treatment revealed no clinically relevant changes. NT-proBNP levels remained within the normal range, which indicated no significant cardiotoxicity (Table 1 and Supplementary Table S4).

## 3 Discussion

These cases presented the clinical use of mitoxantrone hydrochloride liposome replacing conventional doxorubicin in R-CHOP chemotherapy for elderly patients with DLBCL in a real-world setting. All three patients were over 80 years old, presented with poor baseline health status, and exhibited varying degrees of cardiac dysfunction based on heart ultrasound assessments. In addition, one patient had previously received the maximum cumulative dose of anthracyclines. Given these characteristics, considering they might be unable to tolerate conventional doxorubicin-based chemotherapy, mitoxantrone hydrochloride liposome was selected as an alternative. To our knowledge, this is the first report on the use of mitoxantrone hydrochloride liposome-based chemotherapy in combination with rituximab for DLBCL patients aged over 80 years. Notably, all three patients achieved CMR with an acceptable safety profile. These encouraging results underscore the need for future studies involving larger cohorts of elderly patients to further evaluate this therapeutic approach.

DLBCL is most common in the older population and generally has a worse outcome that increases with age, and probably results from a multifactorial origin, such as anthracycline-based therapy-induced cardiotoxic effects, individual frailty caused by loss of bone-marrow function, and the development of unfavorable gene expression profiles (2, 16, 17). Despite the emergence of novel targeted therapies and immunotherapies, these advances often lack sufficient validation in the very elderly (≥80 years), who are frequently underrepresented or excluded from clinical trials. Consequently, there remains no universally accepted standard of care for this population. Moreover, the clinical applicability of modern agents in frail patients is often limited by concerns regarding tolerability and unclear efficacy when combined with standard regimens such as CHOP. In this context, chemotherapy continues to serve as the foundation of treatment for elderly patients with DLBCL (18). We reviewed the treatment approaches and outcomes for elderly patients (aged ≥80 years) with diffuse large B-cell lymphoma (DLBCL) in the literature, based on studies published between 2015 and 2025 in PubMed and Google Scholar (Table 2). The findings indicate that R-miniCHOP (dose-reduced regimens) and R-CHOP–like regimens are commonly employed, while the avoidance or replacement of anthracyclines is considered for patients with a cumulative cardiotoxicity risk (2, 16, 19, 20). Our case report adopted a similar strategy. Instead of conventional anthracyclines, we used mitoxantrone hydrochloride liposome, a synthetic anthracenedione with reduced cardiotoxicity (14, 21). Compared with conventional regimens such as R-miniCHOP, R-CVP, or R-CHOP-like therapies, which report 2/3-year overall survival (OS) rates ranging from 54 to 74% (20, 22) and CR rates between 26.3 and 56.0% (23, 24) in patients aged ≥80 years, the mitoxantrone hydrochloride liposome-based regimen used in our study achieved CMR in all three patients, including one with prior anthracycline exposure and two with baseline cardiac dysfunction. Notably, the treatment was well tolerated, with no observed decline in LVEF or symptomatic cardiac events, in contrast to previously reported rates of cardiac toxicity reaching up to 11% in elderly DLBCL cohorts [e.g., (20)]. The favorable safety profile may be attributed to the liposomal formulations improve drug distribution and reduce systemic toxicity, which enhances tumor targeting while reducing systemic exposure and peak plasma concentrations (8, 9). Prior studies have reported fewer AEs with liposomal mitoxantrone compared to conventional formulations, supporting its use in elderly patients with cardiac comorbidities (13). Although mitoxantrone hydrochloride liposome has demonstrated reduced cardiotoxicity in other malignancies, its application in DLBCL patients aged ≥80 years has not yet been reported. Our case series provides preliminary evidence supporting that mitoxantrone hydrochloride liposome may offer a clinically meaningful alternative for very elderly or anthracycline-ineligible DLBCL patients, meriting further prospective validation.

**Table 2:** Treatment regimen and outcomes for DLBCL patients aged ≥80 years.

Study	Population	Treatment	Outcomes	Cardiotoxicity
Hounsome et al. (22)	DLBCL patients aged ≥80 years (*n* = 2,983)	1 L R-CHOP or R-miniCHOP	3-year OS: 54%	NR
Shi et al. (23)	DLBCL patients aged ≥80 years (*n* = 57)	1 L RD-RCHOP, R-CHOP or R-miniCHOP; 2 L R-GDP, R-ICE; 3 L R-GemOx, lenalidomide	mOS: 28.4 months; 3-year PFS: 68.2% ± 6.9%; ORR: 80%; CR rate: 26.3% (IL)	Fatal cardiac failure 4%
Freudenberger et al. (27)	DLBCL patients aged ≥80 years (*n* = 45)	1 L CHOP	mOS: 7.9 years; mPFS: 1.09 years; ORR: 65%; CR rate: 35%	NR
de Pádua Covas Lage et al. (28)	DLBCL patients aged ≥80 years (*n* = 56)	1 L R-mini-CHOP-21	mOS:3.6 years	NR
Tucci et al. (29)	DLBCL patients aged ≥80 years (*n* = 370)	1 L R-B, R-CVP, chemotherapy without R	> 85 years: 2-year OS and 2-year PFS 48, 43% 80–84 years: 2-year OS and 2-year PFS 63, 56%	NR
Peyrade et al. (24)	DLBCL patients aged ≥80 years (*n* = 120)	1 L ofatumumab and reduced-dose CHOP	2-year OS and 2-year PFS 64·7, 57.2%; ORR, 68%; CR rate: 56%	Grade 3–4 AEs 3%; serious AEs 6%
Choi et al. (30)	DLBCL patients aged ≥80 years (*n* = 194)	1 L R-CHOP, R-CVP, CVP,	mOS:14 years; CR rate: 41.9%	3%
Juul et al. (31)	DLBCL patients aged ≥80 years (*n* = 608)	1 L R ± CHOP, R-CHOEP, CVP	> 85 years: mOS and mPFS 1.9 years, 1.8 years 80–84 years: mOS 2.6 months and mPFS 63%, 1.8 years	NR
Gobba et al. (32)	DLBCL patients aged ≥80 years (*n* = 281)	1 L R-CHOP or R-CHOP–like	mPFS and mOS: 1.3 years and 1.7 years	NR
Oki et al. (20)	DLBCL patients aged ≥80 years with cardiac disease (*n* = 10)	1 L DRCOP	3-year OS 74% for all patients (*n* = 80);	Symptomatic toxicity 4%; asymptomatic LVEF decrease 11% for all patients (*n* = 80)

NR, not reported; 1 L, first line; 2 L, second line; RD-RCHOP, rituximab, doxorubicin, cyclophosphamide, vincristine, and prednisone; R-GDP, rituximab with gemcitabine, dexamethasone and cisplatin; R-ICE, rituximab with ifosfamide, carboplatin and etoposide; R-GemOx, rituximab gemcitabine and oxaliplatin; R-B, rituximab plus either bendamustine; R-CVP, cyclophosphamide, vincristine, and prednisone; DRCOP, rituximab, cyclophosphamide, pegylated liposomal doxorubicin, vincristine, and prednisone.

The dosage was carefully controlled in our study; mitoxantrone hydrochloride liposome was administered at a dose of 10 mg, which corresponds to approximately one-third to one-half of the standard doxorubicin dose used in R-miniCHOP (25 mg/m^2^) (16). This decision was primarily based on the elderly status and tolerability of the patients, regarding the doxorubicin dose in R-miniCHOP, aiming to strike a well-balanced profile between efficacy and safety, with the anticipation of achieving therapeutic benefits without imposing additional strain on the heart. This strategy demonstrated potential clinical value in our study. In addition, our findings suggest the potential efficacy of rituximab plus mitoxantrone hydrochloride liposome-based chemotherapy in biologically high-risk cases. One patient presented with double expression of *MYC* and *BCL2*, classified within the GCB subtype, which is typically associated with an inferior prognosis (25, 26). Despite this, the patient achieved CMR after four cycles of treatment and, as of March 2025, remains in remission. This warrants further investigation in larger studies.

In a review of the literature, we observed that in DLBCL patients aged ≥80 years, Peyrade et al. reported grade 3–4 AEs in 3% of patients and serious AEs in 6% among those receiving reduced-dose CHOP with ofatumumab (24). Similarly, Oki et al. (20) reported a symptomatic cardiotoxicity rate of 4% and an asymptomatic decline in LVEF in 11% of patients (aged >60 years) treated with DRCOP, including a subset of patients with cardiac comorbidities. In comparison with those receiving R-CHOP or R-CHOP-like regimens, our study demonstrated better cardiac safety. No significant changes were observed in LVEF in pre- and post-treatment assessments for the three patients, indicating relative stability in cardiac function throughout the treatment course. Besides, 1 case in our study exhibited mild cardiac dysfunction, with a trend of left ventricular performance decline. However, as this is a case report with a limited sample size, these findings should be interpreted with caution.

Nevertheless, our study has several inherent limitations. First, this case report includes only three patients, and the findings should therefore be interpreted with caution. One patient died during follow-up due to a COVID-19 infection. Although unrelated to the study treatment, this event limited the ability to assess the long-term durability of response. However, the remaining two patients were followed up with for an extended period to provide valuable outcome data. Second, due to the absence of a control group, definitive comparative conclusions cannot be drawn. Further studies involving larger patient cohorts and longer follow-up are needed to confirm these preliminary findings.

## 4 Conclusion

This is the first case report of mitoxantrone hydrochloride liposomal-based chemotherapy plus rituximab treating older patients aged over 80 years with DLBCL, and all patients achieved tumor remission (CMR) after treatment. It is necessary to further increase the number of older patients to investigate the efficacy and safety of this therapy in the future.

## Data Availability

The original contributions presented in the study are included in the article/Supplementary material, further inquiries can be directed to the corresponding author.
