# Supplementary material for: Mitoxantrone hydrochloride liposome-based chemotherapy plus rituximab in elderly patients older than 80 years with diffuse large B-cell lymphoma: case report and review of the literature
Source: Front Med (Lausanne). 2025 Oct 20;12:1629168. doi: 10.3389/fmed.2025.1629168 (PMC12580217; doi:10.3389/fmed.2025.1629168)
Supplement: Supplementary file 1 [file Data_Sheet_1.docx]

**Supplementary Material**

**Table S1. Baseline characteristics**

|  | Case 1 | Case 2 | Case 3 |
| --- | --- | --- | --- |
| Age, years | 81 | 82 | 82 |
| Sex | Female | Female | Male |
| Weight, kg | 42 | 52 | 50 |
| Body surface area, m^2^ | 1.43 | 1.52 | 1.55 |
| KPS score | 80 | 80 | 80 |
| sGA | Healthy | Fit | Fit |
| Ann Arbor stage | Stage IV | Stage III | Stage II |
| IPI | 2 | 2 | 3 |
| GCB | Non-GCB | Non-GCB | GCB |
| B symptoms | None | None | None |
| Bulky disease | None | None | None |
| Extranodal involvement | Bone | None | Ileocecal region |
| Comorbidities | Grade 2 hypertension | None | Type 2 diabetes |

KPS, Karnofsky Performance Status; sGA, simplified geriatric assessment; IPI, International Prognostic Index; GCB, germinal center B-cell.

**Table S2. The change of laboratory values pre- and post-treatment**

|  | Case 1 | |  | Case 2 | |  | Case 3 | |
| --- | --- | --- | --- | --- | --- | --- | --- | --- |
|  | Pre-treatment | Post-treatment |  | Pre-treatment | Post-treatment |  | Pre-treatment | Post-treatment |
| White blood cell, | 2.86 × 10⁹/L | 2.04 × 10⁹/L |  | 3.03 × 10⁹/L | 2.35× 10⁹/L |  | 7.44 × 10⁹/L | 8.26 × 10⁹/L |
| Hemoglobin, g/L | 91 | 80 |  | 104 | 102 |  | 127 | 113 |
| Neutrophils | 2.1× 10⁹/L | 1.51 × 10⁹/L |  | 1.89× 10⁹/L | 1.57 × 10⁹/L |  | 4.7× 10⁹/L | 1.8 × 10⁹/L |
| Platelet | 258× 10⁹/L | 161× 10⁹/L |  | 144× 10⁹/L | 111× 10⁹/L |  | 294× 10⁹/L | 188× 10⁹/L |

**Table S3. Summary of adverse event**

|  | Case 1 | Case 2 | Case 3 |
| --- | --- | --- | --- |
| Anemia | Grade 2 | Grade 2 | Grade 2 |
| Neutrophil count decreased | Grade 2 | Grade 2 | Grade 2 |
| Gastrointestinal symptoms | Grade 2 | grade 2 | - |
| Peripheral nerve infection | Grade 1 | - | - |
| Myelosuppression | Grade 1 | Grade 1 | - |

**Table S4.** **The change in** **echocardiogram data pre- and post-treatment**

|  | Case 1 | |  | Case 2 | |  | Case 3 | |
| --- | --- | --- | --- | --- | --- | --- | --- | --- |
|  | Pre-treatment | Post-treatment |  | Pre-treatment | Post-treatment |  | Pre-treatment | Post-treatment |
| Anteroposterior dimension of left ventricle, mm | 27 | 24 |  | 26 | 31 |  | 26 | 27 |
| Left ventricular diastolic dysfunction, mm | 39 | 42 |  | 48 | 48 |  | 32 | 46 |
| Stroke volume, mL | 42 | 55 |  | 70 | 68 |  | 29 | 60 |
| Fractional shortening, % | 37 | 38 |  | 37 | 33 |  | 42 | 33 |
| Aortic valve, cm/s | 13 | 10 |  | 120 | 139 |  | 100 | 137 |
| Mitral E velocity, cm/s | 85 | 100 |  | 69 | 74 |  | 47 | 60 |
| Mitral E′ velocity, cm/s | 5.2 | 7.1 |  | 3.8 | 4.4 |  | 6.5 | 8.1 |
| E/A ratio | 0.7 | 0.7 |  | 0.6 | 0.8 |  | 0.7 | 0.6 |
| E/E′ ratio | 16.2 | 14.2 |  | 18.4 | 11.8 |  | 7.3 | 7.4 |
| Cardiac output, L/min | 4.3 | 3.4 |  | 5.9 | 5.6 |  | 2.6 | 4.5 |
| Left ventricular ejection fraction, % | 61 | 68 |  | 66 | 62 |  | 74 | 62 |
